# Nitric Oxide, Iron and Neurodegeneration

**DOI:** 10.3389/fnins.2019.00114

**Published:** 2019-02-18

**Authors:** Chao Liu, Mui Cheng Liang, Tuck Wah Soong

**Affiliations:** ^1^Department of Physiology, Yong Loo Lin School of Medicine, National University of Singapore, Singapore, Singapore; ^2^Jiangsu Province Key Laboratory of Anesthesiology, Xuzhou Medical University, Xuzhou, China; ^3^Jiangsu Province Key Laboratory of Anesthesia and Analgesia Application Technology, Xuzhou Medical University, Xuzhou, China; ^4^Neurobiology/Ageing Program, Centre for Life Sciences, National University of Singapore, Singapore, Singapore; ^5^National Neuroscience Institute, Singapore, Singapore

**Keywords:** oxidative stress, Parkinson’s disease, nitric oxide, iron homeostasis, S-nitrosylated proteins

## Abstract

Iron is a crucial cofactor for several physiological functions in the brain including transport of oxygen, DNA synthesis, mitochondrial respiration, synthesis of myelin, and neurotransmitter metabolism. If iron concentration exceeds the capacity of cellular sequestration, excessive labile iron will be harmful by generating oxidative stress that leads to cell death. In patients suffering from Parkinson disease, the total amount of iron in the substantia nigra was reported to increase with disease severity. High concentrations of iron were also found in the amyloid plaques and neurofibrillary tangles of human Alzheimer disease brains. Besides iron, nitric oxide (NO) produced in high concentration has been associated with neurodegeneration. NO is produced as a co-product when the enzyme NO synthase converts L-arginine to citrulline, and NO has a role to support normal physiological functions. When NO is produced in a high concentration under pathological conditions such as inflammation, aberrantly S-nitrosylated proteins can initiate neurodegeneration. Interestingly, NO is closely related with iron homeostasis. Firstly, it regulates iron-related gene expression through a system involving iron regulatory protein and its cognate iron responsive element (IRP-IRE). Secondly, it modified the function of iron-related protein directly via S-nitrosylation. In this review, we examine the recent advances about the potential role of dysregulated iron homeostasis in neurodegeneration, with an emphasis on AD and PD, and we discuss iron chelation as a potential therapy. This review also highlights the changes in iron homeostasis caused by NO. An understanding of these mechanisms will help us formulate strategies to reverse or ameliorate iron-related neurodegeneration in diseases such as AD and PD.

## Introduction

Iron, one of the most abundant metals in the earth crust (Weber et al., 2006), is a transition metal. Iron has the ability to dynamically form compounds with organic ligands, while switching between Fe^2+^ (ferrous) and Fe^3+^ (ferric) state. Because of this property, iron has a crucial role in catalyzing electron transfer (redox) in certain enzymatic reactions. It is critical in cellular respiration, oxygen transport, and many other biological reactions (Aisen et al., 2001). For example, deficiency of iron has led to hypomyelination in some children (Oski et al., 1983; Lozoff et al., 2006). Iron-mediated production of free radicals via the conversion of hydrogen peroxide can damage many cellular structures. Most cellular iron are bound as iron–sulfur (Fe–S) clusters and by heme proteins. Cytoplasmic ferrous iron can be found in a labile and chelatable state that is known as the labile iron pool (also termed “free iron”). It is thought to be the main contributor of oxidative stress during iron overload (Piloni et al., 2016). Iron in the labile pool can be loosely bound to peptides, carboxylates and phosphates as compounds with low-mass, while some might exist as hydrated free iron. In mammalian cells, the labile iron concentration is less than 1 μM, and less than 5% of total iron (Kakhlon and Cabantchik, 2002). The labile iron exerts its toxicity by generating reactive oxygen species. Fe^2+^ catalyzed reactions produce free radicals via the Fenton’s reaction:

$${Fe}^{2+}+H_{2}O_{2}\to {Fe}^{3+}+HO•+{OH}^{-}$$

$${Fe}^{3+}+H_{2}O_{2}\to {Fe}^{2+}+HOO•+H^{+}$$

On the other-hand, labile iron taken up by the mitochondrial mitoferrin can be incorporated into Fe–S clusters and heme groups (Hentze et al., 2010). Cellular oxidative stress induced by iron overload is characterized by increased lipid peroxidation and protein and nucleic acid modifications (Ward et al., 2014). These free radicals interact with the surroundings indiscriminately. In the brain, iron plays a role in other enzymatic reactions that produce neurotoxins. The cells which have active iron metabolism are more prone to iron toxicity, such as dopaminergic neurons, which need iron for dopamine metabolism (Hare and Double, 2016). MRI imaging showed that iron deposition was observed in the putamen, globus pallidus, red nucleus and substantia nigra, but yet not all of them have dramatic neuronal loss in PD patients. The major neuronal loss in PD happens in dopaminergic neurons of the substantia nigra pars compacta, which is unique because dopamine metabolism produces several neurotoxins. These toxins are more harmful than widespread oxidative stress caused by iron deposition (Zhou et al., 2010).

Inflammation is commonly observed in the brains of animal models or patients with neurodegenerative diseases like AD and PD (Block and Hong, 2005). Neuroinflammation can produce a variety of proinflammatory factors including TNF-α, IL-6 and nitric oxide (NO). At low levels, NO supports normal neuronal functions via S-nitrosylation of target proteins. However, when NO is produced in a high concentration under pathological conditions such as inflammation, aberrantly S-nitrosylated proteins can initiate neurodegeneration (Nakamura et al., 2013).

We discuss in this review how iron homeostasis is affected in neurodegenerative disease, especially by NO, and how dysregulation of iron can lead to neurodegeneration.

## Iron Homeostasis

About half of the total iron is contained in hemoglobin which is needed to transport oxygen along the blood-stream. The other half is, however, stored in complex with ferritin in various cells, mostly in bone marrow, liver and spleen. Only a small portion is circulated in the plasma, bound to transferrin (Conrad and Umbreit, 2000). The absorption of dietary iron takes place in the duodenum. The iron is absorbed by enterocytes of the duodenal lining bound by a protein, such as heme protein, or through divalent metal transporter 1 (DMT1) in ferrous form. The lining cells of the intestine can either bind iron by ferritin for storage, or release it to the body via ferroprotein, the only known mammalian iron exporter (Gropper et al., 2013). Ferroportin (Fpn) can be post-translationally repressed by a peptide hormone of a 25-amino acid length, named hepcidin, Hepcidin binds to Fpn and internalizes it within the cell (Nemeth et al., 2004). Fpn expression can be modulated by the IRP-IRE regulatory mechanism. If the iron concentration is too low, the IRP-IRE binding increases, thus inhibiting Fpn translation. On the other hand, Fpn translation can also be regulated by the microRNA, miR-485-3p (Sangokoya et al., 2013). The ferroxidase, hephaestin, found in the small intestine, oxidizes Fe^2+^ to Fe^3+^ and therefore helps Fpn transfer iron across the small intestine basolateral membrane (Ward et al., 2014). Iron metabolism in the body is thus regulated by regulating each of these steps. For instance, under iron-deficient anemic conditions, enterocytes will produce higher levels of Dcytb, DMT1 and transferrin receptor (TfR) proteins (Fleming and Bacon, 2005). Absorption of dietary iron is also enhanced by vitamin C and, in contrast, reduced by excess calcium, zinc, or manganese.

**FIGURE 1:**
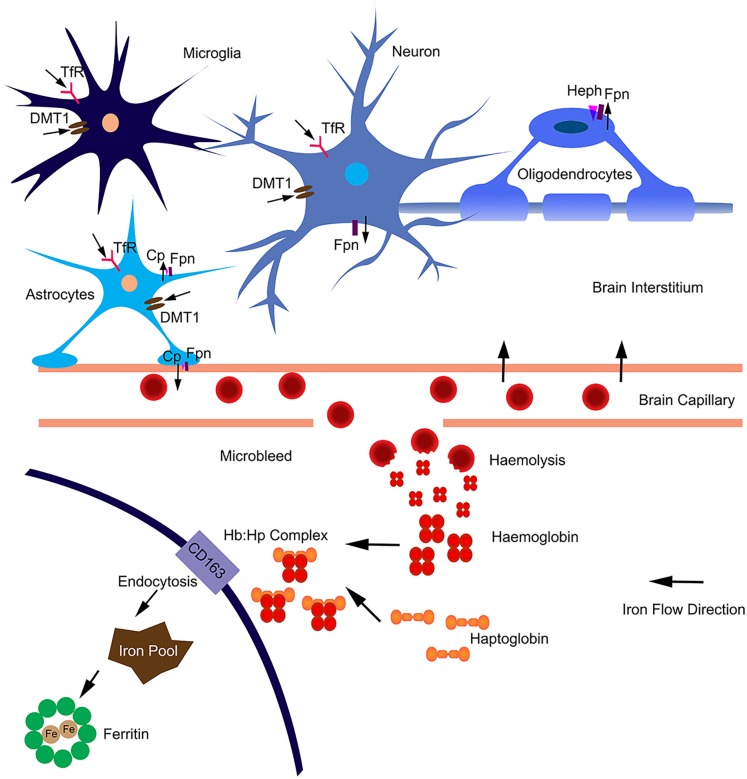
Iron homeostasis in the brain. The iron was uptake from blood through brain capillary epithelial cells. Neurons express TfR, DMT1 and Fpn; absorb iron from brain interstitium and export excess iron. Astrocytes form intimate contact with brain capillary epithelial cells through end-feet and may influence iron transport between blood and brain. Astrocytes express TfR1, DMT1 and ZIP-14; and efflux iron via Fpn partnered with ceruloplasmin (Cp). Astrocytes may also efflux iron via endothelial cells into blood. Microglia can acquire iron via TfR1, DMT1 from brain intermedium. Microglia are unable to efflux iron as Fpn is internalized from the cell surface. In aged or neurodegenerative brain, microbleeds may occur. Hemoglobin is released after haemolysis. Haptoglobin binds with hemoglobin form a tight complex. The Hb:Hp complex is bound with the receptor CD163 and undergo endocytosis. The complex are degraded after endocytosis and the iron is absorbed into intracellular iron pool and stored in ferritin. The iron flow direction is indicated by black arrows.

In the brain, iron uptake is through the blood-brain barrier (Figure 1). Iron is taken up by the capillary endothelial cell TfR, in the form of transferrin-Fe^3+^ (Tf-Fe^3+^). The iron is transported to cerebral compartment from the basolateral membrane of endothelial cells, and is then made available to neurons and glia. Of note, oligodendrocytes stained for most of the detectable iron in the brain. Transferrin is also found predominantly in these cells (Bloch et al., 1985), which is important for myelination formation. Oligodendrocytes support myelin membranes that are many times of the weight of their somata, and need to generate a lot of ATP (Cammer, 1984), hence they need a lot of iron to maintain their metabolism. The TfR is expressed in blood vessels, and in cortical, striatal and hippocampal neurons (Connor and Menzies, 1995). Most types of cells uptake iron primarily through transferrin-mediated endocytosis, via TfR1, TfR2, and GAPDH (West et al., 2000; Kumar et al., 2011; Figure 1). Fe^3+^ is dissociated from transferrin in the endosome, reduced to Fe^2+^ by a STEAP family reductase, and exported from endosome into cytosol by DMT1 (Hentze et al., 2010). Alternatively, Fe^2+^ can be absorbed directly via the plasma membrane divalent cation transporters such as DMT1 and ZIP14 (Lane et al., 2015). Fpn is the only protein know for iron efflux so far (Zecca et al., 2004). Neurons and astrocytes express TfR, DMT1, and Fpn (Moos et al., 2007; Zarruk et al., 2015). The astrocyte end feet form intimate contact with brain capillary epithelial cells and play an important role to mediate iron export from blood to brain (Moos et al., 2006). Astrocytes express ceruloplasmins which oxidize Fe^2+^ to Fe^3+^ and facilitate iron export from astrocytes (Figure 1). During brain inflammation, iron is accumulated in microglia stead of astrocyte. The efflux mechanism of iron in microglia was disrupted in brain inflammation because Fpn is endocytosed, induced by hepcidin, and microglia cells do not express ferroxidase (Zarruk et al., 2015). In contrast, astrocytes do not accumulate iron, showing robust expression of influx and efflux proteins including Fpn and ferroxidase, with normal iron recycling capability (Figure 1). Ferritin, a major iron storage protein in the brain, compromises the H- or L-ferritin monomers. Neurons and oligodendrocyte express H-ferritin, which have a high iron metabolite rate. Microglia express L-ferritin, which is associated with iron storage (Connor et al., 1994). Although oligodendrocytes have the highest iron accumulation in normal aging brain, there is little effect on myelination and oligodendrocyte in neurodegenerative diseases. Oligodendrocytes have high levels of ferritin expression (Connor et al., 1994), which provide more buffering capacity for free iron and therefore affording more protective effect. Fpn needs to couple with ferroxidase to export iron. Astrocytes express ceruloplasmin, while oligodendrocytes express another ferroxidase, hephaestin, to facilitate iron export from oligodendrocyte (Schulz et al., 2011). Oligodendrocytes down regulate TfR1 to reduce iron uptake after maturation (Han et al., 2003). These iron level-regulating mechanisms protect oligodendrocytes in neurodegenerative diseases. The iron absorbed from blood during different age is more than what is accumulated in the brain, indicating that there is a iron efflux mechanism in the brain. The iron export from brain to blood maybe through the cerebrospinal fluid (Moos et al., 2007).

In addition, the highest iron load in the brain is seen in relation to chronic microbleeds. Cerebral microbleeds are likely to be caused by cerebral atherosclerosis in aged people or people with neurodegenerative diseases (Martinez-Ramirez et al., 2014). The iron from hemolysis of erythroid cells may be the major source of iron load in aged or neurogenerative brain (Figure 1). Erythroid cells contain most of the body iron in hemoglobin (Hb). Hb contains 70% of the total body iron in its heme moiety. Hb is not restricted in erythroid cells, but expressed in glia, macrophage and neurons. Hb is an oxygen reservoir inside the cell and regulate mitochondria function. While Hb mainly exists intracellularly, it will be released extracellularly during hemolysis. Haptoglobin (Hp) is mostly produced by hepatic cell and binds free Hb in plasma with very high affinity (Wicher and Fries, 2010). In the brain, Hp is produced locally by oligodendrocyte (Zhao et al., 2009). The Hb–Hp complex has a membrane receptor CD163. The interaction between them leads to internalization of CD163 (Figure 1), Hb dissociating from Hp, Hb heme degradation, and iron being absorbed into the intracellular iron pool. Following intravascular hemorrhage, hippocampal and cortical neurons express CD163 in the brain (Garton et al., 2016), which facilitates iron absorption, post-hemolysis.

Although rodent models are widely used for iron study, it is noteworthy that iron accumulation and cellular storage are different between humans and experimental rodent models. Rodent generally have a lower basal level of iron deposition in the brain, especially in young animals (less than one year old), as shown by several groups (Jeong and David, 2006; Liu et al., 2018).

## Iron Deposition Changes in AD and PD

In AD, iron hemostasis is disrupted. Transferrin has been shown to be decreased consistently, and in particular in the white matter, White matter is thought to play a major role in neurodegeneration, and increased peroxidative damage to white matter is known to take place in AD (Connor et al., 1992). High concentration of iron is accumulated in Aβ plaques and tau tangles which is characteristic of AD. The Aβ plaques contain a fairly large amount of labile iron, while the neighboring cells express significant levels of ferritin and transferrin receptors (Connor et al., 1995). Moreover, iron influence APP translation via IRP-IRE system. There is an IRE within the 5′-UTR of APP mRNA (Rogers et al., 2002), and in response lowered intracellular iron as a result of chelation by desferrioxamine and clioquinol, translation of APP was selectively down-regulated. APP interacts with Fpn, and it has ferroxidase activity as it possessed the conserved H-ferritin-like active site, and Zn^2+^ can specifically inhibit this active site (Duce et al., 2010; Bush, 2013). Lei et al. showed that tau deficiency caused iron accumulation in brain and dopaminergic neuron degeneration, which led to parkinsonism in mice with dementia. Tau promotes the export of neuronal iron by facilitating the trafficking of APP to the plasma membrane. The study suggested that Alzheimer disease, Parkinson disease and tauopathies that are associated with the iron toxicity due to the loss of soluble tau could in principle be rescued by a pharmacological agent such as clioquinol, an iron chelator (Lei et al., 2012).

Several studies reported that iron deposition was increased in the substantia nigra according to the severity of the disease in PD patients (SN) (Dexter et al., 1987, 1989; Hirsch et al., 1991). The researchers used plasma spectroscopy to detect iron concentration quantitatively in various brain regions (Dexter et al., 1989). In PD brain, histology studies showed that iron accumulate in neurons and glia in SN (Jellinger et al., 1990). *In vitro*, the formation of α-synuclein fibrils can be accelerated by the presence of Fe^3+^ (Uversky et al., 2001) and it was also reported that redox-active iron was sequestrated by Lewis bodies in SN (Castellani et al., 2000). Furthermore, there are reports that a dysfunction in the IRP-IRE system that results in iron accumulation gave rise to α-Syn-induced toxicity (Li et al., 2010; Febbraro et al., 2012), that led to PD pathogenesis (Rocha et al., 2017). Similarly, in almost all PD patient brains the Lewy bodies contained aggregated α-Syn (Wakabayashi et al., 2007).

The reason for iron accumulation in SN is unclear. Several hypotheses proposed include increased brain-blood-barrier (BBB) permeability (Faucheux et al., 1999; Kortekaas et al., 2005), increased neuroinflammation (Block and Hong, 2005; Conde and Streit, 2006; Machado et al., 2011), increased DMT1 isoform expression or function (Block and Hong, 2005; Salazar et al., 2008; Machado et al., 2011; Liu et al., 2018), and altered transferrin or lactoferrin transport (Faucheux et al., 1995; Mastroberardino et al., 2009). Cellular iron accumulation in PD brain may be caused by elevated influx or decreased efflux. Inflammation could contribute to iron accumulation by either increasing DMT1 uptake activity or TfR transport activity. In a mouse model, DMT1 activity was increased to mediate the iron uptake (Salazar et al., 2008), and this increase may be due to direct S-nitrosylation of DMT1 (Liu et al., 2018).

## No Production in Immune Response and Neurodegeneration

NO is a gaseous signaling molecule that initially was thought as a dilator in blood vessels, with guanylyl cyclase as the major effector. NO binds to the heme group of guanylyl cyclase and activates it in the presence of iron. High cGMP level are associated with release of neurotransmitters including glutamate, acetylcholine and glycine. NO participates in tumor and bacteria immunity and in the central nervous system, it acts as a retrograde neurotransmitter. In the nervous system, NO has both physiological and pathological functions. For example, NO contributes to long-term potentiation (LTP) and long-term depression (LTD), and thus it plays a role in learning and memory (Schuman and Madison, 1991; Shibuki and Okada, 1991; Lev-Ram et al., 1997; Kakegawa and Yuzaki, 2005; Kakizawa et al., 2012). NO enhances CREB expression to mediate the response to brain-derived neurotrophic factor (Riccio et al., 2006). NO also mediates glutamate-NMDA receptor (NMDAR) signaling as glutamate activates NMDAR in neurons, leading to Ca^2+^ influx, and activation of nNOS (Xu et al., 2018). The synaptic NMDARs mediate neuroprotection, while the extrasynaptic NMDARs mediate neurodegeneration (Talantova et al., 2013; Molokanova et al., 2014), implicating the diverse signaling pathways triggered by NO. NO also binds to other iron-containing proteins, such as mitochondrial aconitase. Mitochondrial aconitase has a [4Fe-4S]^2+^ cluster, which is a possible target for NO-induced toxicity (Drapier and Hibbs, 1986, 1988). The interaction between mitochondrial aconitase and superoxidase are the major cause to mitochondrial damage (Vasquez-Vivar et al., 1999). Most of the cytotoxicity of NO is attributed to the production of peroxynitrite (Pacher et al., 2007), which is more powerful to produce radicals. Peroxynitrite is produced from the diffusion-controlled reaction between NO and superoxide *in vivo* (Squadrito and Pryor, 1995). Peroxynitrite is a strong oxidant and it interacts with electron-rich groups, including Fe–S cluster. It is reported that peroxynitrite is far more effective to produce hydroxyl radicals than Fenton’s reaction (Beckman et al., 1990; Darley-Usmar et al., 1992; Hogg et al., 1992). Peroxynitrite is an important intermediator for protein nitration and oxidation, lipid peroxidation, mitochondria dysfunction, and finally causes apoptosis and necrosis (Radi, 2018).

NO is produced by NOS through the conversion of L-arginine to citrulline. Three distinct NOS isoforms have been identified in the brain (Forstermann et al., 1991). Neuronal NOS (nNOS) is expressed in neurons, while endothelial (eNOS) is expressed in brain endothelial cells. They are Ca^2+^/calmodulin-dependent and synthesize NO in a short period in response to receptor activation or extracellular stimuli (Moncada et al., 1991). Inducible NOS (iNOS) is expressed in glia cells upon brain injury or inflammation. Inducible NOS produces a large amount of NO upon stimulation by proinflammatory cytokines over a long period of time (Green et al., 1994).

In human immune response, NO is produced by phagocytes such as monocytes, macrophages, and neutrophils. In phagocytes, interferon-gamma (IFN-γ) or tumor necrosis factor (TNF) activates iNOS (Green et al., 1993). On the other hand, transforming growth factor-beta (TGF-β), interleukin-4 (IL-4) or IL-10 weakly inhibits iNOS. As such, phagocytes contribute to inflammatory and immune responses via NO (Green et al., 1994). In an immune response, NO is secreted as free radicals that is toxic to intracellular pathogens. The modes of action are via DNA damage (Wink et al., 1991; Nguyen et al., 1992) and degradation of Fe–S centers into iron ions and iron-nitrosyl compounds (Hibbs et al., 1988). The molecular effects of NO depend on two kinds of reactions: S-nitrosylation of thiols and the nitrosylation of some metalloenzymes. Guanylate cyclase, a NO activated heme-containing enzyme, is an essential component of the relaxing function of NO on smooth muscles (Derbyshire and Marletta, 2009). cGMP activates protein kinase G that lead to the re-uptake of Ca^2+^ and the rise in cytoplasmic Ca^2+^ activates calcium-activated potassium channels triggering the relaxation of smooth muscle (Rhoades and Tanner, 2003).

In addition to neuro-inflammatory stimuli, induction of iNOS expression in astrocytes, macrophages, and microglia by Aβ oligomers or by toxins such as 1-methyl-4-phenyl-1,2,3,6-tetrahydropyridine (MPTP) have been reported to increase NO levels in the degenerating brain (Liberatore et al., 1999; Medeiros et al., 2007; Nakamura et al., 2013). Knockdown of iNOS in APP/PS1 AD mouse model ameliorate AD-related symptom including Aβ plaque formation, premature death, astroglioses and microgliosis (Nathan et al., 2005). However, in the Tg2576 APP AD mouse model, ablation of iNOS exacerbated spatial learning and memory and tau pathology, providing evidence that NO may have a neuroprotective role (Wilcock et al., 2008).

## No Regulation on Iron Homeostasis

**FIGURE 2:**
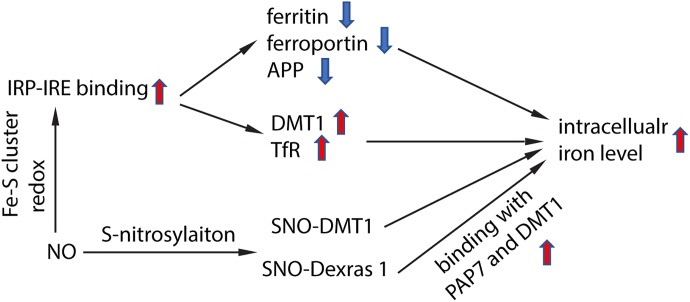
Schematic chart showing how NO regulate iron homeostasis. NO regulates IRP-IRE binding through redox reaction with Fe–S cluster in IRP, hence regulates the transcription of iron-metabolism-related proteins, and elevates intracellular iron level. NO also directly S-nitrosylates DMT1, which enhances DMT1 transporter function. In addition, NO S-nitrosylates Dexras 1 and enhances the binding of Das1-PAP7-DMT1 complex and finally enhances iron uptake.

**FIGURE 3:**
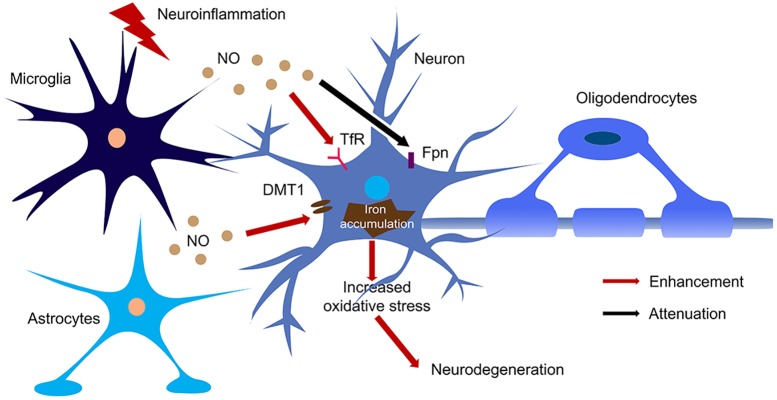
The regulation of NO on iron homeostasis in the brain during neuroinflammation. Large amount of NO was produced by microglia and astrocytes upon activation of iNOS during neuroinflammation. NO enhanced the translation of TfR and DMT1 and decreased the translation of Fpn, hence increased iron accumulation in neurons. The iron accumulation leads to oxidative stress and finally caused neurodegeneration.

NO targeted proteins have been partially characterized. NO can interact with Fe–S cluster containing protein and influence their enzyme activity. One of the Fe–S cluster-containing proteins is IRE-binding protein (also termed “iron regulatory protein,” IRP). Cytosolic iron concentrations sensed by IRPs could post-transcriptionally adjust the expression of iron metabolizing genes to optimize the availability of labile iron. IRPs bind to iron-responsive elements (IRE), which are specific non-coding mRNA sequences, to control iron metabolism. IREs are of 30 nucleotide in length found along RNA motifs, and they contain the CAGUGN sequence (the classic IRE motif) that form a stem-loop structure (Molokanova et al., 2014). IREs are found either within the 3′-UTR (untranslated region) or 5′-UTR regions of a specific mRNA. IRP1 and IRP2 are examples of two RNA-binding proteins that interact with IRE to modulate the translation of either the ferritin or Fpn mRNA, and they also control the stability of TfR and DMT1 mRNAs. The binding of IRPs and IREs is regulated by free iron concentration. Therefore, IRPs can act as either a translation enhancer or inhibitor (Pantopoulos, 2004; Piccinelli and Samuelsson, 2007). As ferritin and Fpn transcripts contain IRE in their 5′-UTRs, their translation will be inhibited by IRPs when there is iron deficiency (Hentze et al., 2010). The decreased expression of ferritin and Fpn reduces free iron binding and export, leading to an increased in availability of labile iron for use by the cell. In contrast, the TfR and DMT1 transcripts contain 3′-UTR IREs that bind IRPs, and when cytoplasmic iron is deficient, the stabilization of transcripts will increase synthesis of TfR and DMT1 proteins to enhance iron uptake (Hentze et al., 2010). Examples of transcripts that contain IREs include those that encode the ferritin subunits L and H, TfR, Fpn, DMT1, mitochondrial aconitase, succinate dehydrogenase, erythroid aminolevulinic acid synthetase, amyloid precursor protein and a-synuclein. These downstream genes suggest that iron has close regulation of iron metabolism, redox and neurodegeneration via the IRP-IRE system. NO could regulate IRP-IRE binding, which in turn regulates many iron metabolism-related proteins (Figure 2). NO acts on DMT1 by increasing IRP1 binding to the IRE sequence located at the 3′-UTR, to stabilize and increase DMT1 transcript level, akin to cells exposed to low iron condition (Weiss et al., 1993; Jaffrey et al., 1994; Wallander et al., 2006; Skjorringe et al., 2015). Similarly, NO can regulate ferritin, Fpn and TfR via regulating the interaction of IRP-IRE binding, and hence regulate iron metabolism (Figure 3). Another report also showed that NO can enhance iron deposition in the brain via decreasing APP expression (Ayton et al., 2015). The authors reported obvious decrease in expression of APP in substantia nigra of PD brain. APP KO mice have iron-dependent dopaminergic neuron loss, while APP overexpressing mice have protection effect in MPTP mouse model, as APP facilitates iron efflux. NO decreases APP expression via the IRP-IRE system and this may explain how NO leads to dopaminergic neuron loss in PD.

NO also regulates iron metabolism-related proteins in other ways, such as S-nitrosylation (Figure 2). Another pathway to increase iron uptake via DMT1 is by S-nitrosylation of Dexras 1 to indirectly regulate DMT1 function and enhance Fe^2+^ uptake as reported in PC12 and cortical neurons (Cheah et al., 2006). Recently, we have shown that NO directly modulated DMT1 and enhanced its function via S-nitrosylation. This is unexpected as S-nitrosylation of proteins important in PD such as Parkin and XIAP resulted in compromised functions. Besides, many S-nitrosylated proteins have been identified in the past decade, and of note, those that have been functionally characterized have a loss-of-function (Nakamura et al., 2013). In this regard, enhanced DMT1 activity arising from S-nitrosylation would therefore present a mechanism by which Fe^2+^ could be accumulated over aging and contribute to age-dependent neurodegeneration (Figure 3).

## Potential Therapy for Neurodegenerative Diseases Targeted to Iron Deposition

The potential therapeutic use of iron chelators gained much attention in recent years (Ward et al., 2014; Zhou and Tan, 2017). The strategy to target iron deposition is either to chelate iron directly or to regulate iron homeostasis, including NO-regulated iron absorption. The candidate compounds should be BBB-permeant and easily penetrate cell membrane, chelate free iron and minimize the side effect to normal iron metabolism.

Several iron chelators were used to deplete excessive iron and yielded promising clinical outcome. The clinical trial using deferiprone at 30 and 20 mg/kg per day was carried out in PD patient, and the compound was well-tolerated. The syndrome was improved and the iron content in SN significantly decreased as monitored by MRI (Devos et al., 2014). Deferiprone seems quite promising so far, and is waiting to be tested in further clinical trial in larger population. Furthermore, in several *in vivo* PD models, iron chelators, including deferasirox, deferrioxamine, VAR10303 and D-607, have been used to significantly attenuate DA neuronal loss (Ghosh et al., 2010; Dexter et al., 2011; Bar-Am et al., 2015; Das et al., 2017).

Desferrioxamine has been shown to decelerate AD progression (Rogers and Lahiri, 2004). However, as DFO is unstable with poor BBB permeability (Bandyopadhyay et al., 2010), clioquinol, a BBB-permeant iron chelator, was used instead in clinic to mitigate cognitive loss by reducing plasma Aβ levels in AD patients (Ritchie et al., 2003). As clioquinol has side effects associated with myelopathies (Zhang et al., 2013), PBT2, a second-generation 8-hydroxyquinoline analog metal chaperone which can bind transition metal, was used in clinical trial for AD patients. The patients who received 250 mg/kg PBT2 have substantially reduced Aβ level in cerebral spinal fluid and showed cognitive improvement in a Phase II trial (Lannfelt et al., 2008; Faux et al., 2010). However, in another Phase II trial announced by the Australian company Prana Biotechnoloy in 2014, PTB2 failed to improve brain amyloid deposition, neuronal function, brain atrophy and cognition in a one-year course treatment. Several other iron chelators, such as VK28 (Fe^3+^ chelator), HLA20 and M30 (Fe^3+^ chelator with N-propargylamine-like properties), can not only suppress APP expression but also reduce Aβ level in the brain (Bandyopadhyay et al., 2010).

Besides direct chelation of iron, potent nontoxic IRE inhibitors with excellent BBB penetrating capacity were also thought to have high therapeutic significance in neurodegenerative diseases. The IRE inhibitors that down-regulate translation of APP and α-Syn and prevent protein aggregation can support survival of neurons. To date, a few promising drug candidates of IRE inhibitors have been characterized and are being tested in various clinical trials for AD and PD patients (Zhou and Tan, 2017). Posiphen is a natural product that has been shown to inhibit translation of both APP and a-synuclein proteins. Furthermore, it is nontoxic and potent (Rogers et al., 2011). The inhibitory effect has been validated by various experiments done *in vitro* and *in vivo* (Lahiri et al., 2007; Yu et al., 2013). A phase I human clinical trial in subjects with mile cognitive impairment have shown that posiphen lowered APP production by 50 % in CSF and was well-tolerated (Maccecchini et al., 2012). The compound is still waiting for further clinical trials to test the efficacy for AD and PD (Bandyopadhyay et al., 2010). Another compound JTR-009, screened from a 110,000-compound library, was identified to have a more potent effect on APP translation than posiphen. JTR-009 is thought to bind to 5′-UTR IRE directly and selectively inhibit APP expression, which was validated in SH-SY5Y cells (Bandyopadhyay et al., 2013). Another case for targeting iron homeostasis is inhibiting DMT1 function. To inhibit NO-mediated DMT1 functional increase, we used the NOS inhibitor L-name to reduce NO-mediated iron deposition in LPS-evoked mouse inflammatory model. L-NAME significantly ameliorated SN dopaminergic neuron loss and LPS-induced behavior deficit (Liu et al., 2018).

## Conclusion and Future Perspectives

Iron homeostasis is elaborately regulated in the human brain, and iron accumulation is closely associated with neurodegenerative diseases. NO regulates iron deposition at several levels. So far, therapeutic targeting of iron deposition has yielded some promising results, and further clinical trials in larger populations are still needed. There are still some questions that remained unresolved. For example, how iron is transported through brain capillary epithelial cells and more specifically, how S-nitrosylation of DMT1 enhanced its transporter activity. The technique for iron and NO concentration detection is a limitation, especially for accurate detection of Fe^2+^ and Fe^3+^ concentrations. A real-time, quantitative and *in vivo* detection technique will be extremely valuable for the field.

## Author Contributions

CL and MCL drafted the manuscript. TWS critically edited the manuscript. All authors approved the final version of the manuscript.

## Conflict of Interest Statement

The authors declare that the research was conducted in the absence of any commercial or financial relationships that could be construed as a potential conflict of interest.
